# Rheumatoid Arthritis is Not Associated with Increased Inpatient Mortality in Patients Admitted for Acute Coronary Syndrome

**DOI:** 10.7759/cureus.9799

**Published:** 2020-08-17

**Authors:** Ehizogie Edigin, Hafeez Shaka, Precious Eseaton, Shakeel Jamal, Asim Kichloo, Pius E Ojemolon, Iriagbonse Asemota, Emmanuel Akuna, Augustine Manadan

**Affiliations:** 1 Internal Medicine, John H. Stroger, Jr. Hospital of Cook County, Chicago, USA; 2 Internal Medicine, University of Benin, Benin City, NGA; 3 Internal Medicine, Central Michigan University College of Medicine, Saginaw, USA; 4 Internal Medicine, Central Michigan University, Saginaw, USA; 5 Anatomical Sciences, St. George's University, St. George's, GRD; 6 Rheumatology, John H. Stroger, Jr. Hospital of Cook County, Chicago, USA

**Keywords:** rheumatoid arthritis, cardiovascular diseases, rheumatic diseases, disease modifying anti-rheumatic drugs, hospitalization, mortality

## Abstract

Objectives: This study aims to compare the outcomes of patients admitted primarily for acute coronary syndrome (ACS) with and without a secondary diagnosis of rheumatoid arthritis (RA).

Methods: Data were abstracted from the National Inpatient Sample (NIS) 2016 and 2017 Database. The NIS was searched for hospitalizations of adult patients with ACS as principal diagnoses, with and without RA as a secondary diagnosis. The primary outcome was inpatient mortality. Secondary outcomes were hospitalization characteristics and cardiovascular therapies. Multivariate logistic and linear regression analysis were used accordingly to adjust for confounders.

Results: There were over 71 million discharges included in the combined 2016 and 2017 NIS database. Out of 1.3 million patients with ACS, 22,615 (1.7%) had RA. RA group was older (70.4 vs 66.8 years, P<0.001) as compared to the non-RA group, and had more females (63.7% vs 37.7%, P<0.0001). Patients with RA had a 16% reduced risk of in-hospital mortality: odds ratio (OR) 0.84, 95% confidence interval (CI) (0.72-0.99), P=0.034; less odds of undergoing intra-aortic balloon pump (IABP): OR 0.78, 95% CI (0.64-0.95), P=0.015; and 0.18 days shorter hospital length of stay (LOS): 95% CI (0.32-0.05), P=0.009. However, odds of undergoing percutaneous coronary intervention with drug-eluting stent (PCI DES) at OR 1.14, 95% CI (1.07-1.23), P<0.0001 was significantly higher in the RA group compared to ACS without RA.

Conclusions: Patients admitted for ACS with co-existing RA had lower adjusted inpatient mortality, less odds of undergoing IABP, shorter adjusted LOS, and greater adjusted odds of undergoing PCI DES compared to those without RA.

## Introduction

Rheumatoid arthritis (RA) is a debilitating, chronic autoimmune disorder that has both articular and extra-articular manifestations with marked systemic inflammation [1,2]. Acute coronary syndrome (ACS) refers to any group of clinical symptoms consistent with acute myocardial ischemia and includes ST-segment elevation myocardial infarction (STEMI), non-ST-segment elevation myocardial infarction (NSTEMI), and unstable angina (UA) [3].

Cardiovascular (CV) diseases are the leading cause of mortality and morbidity among patients with RA [4-6]. These patients are at increased risk compared to patients without coexisting RA due to chronic systemic inflammation [7-11]. The effect of this increased CV risk on outcomes of ACS in RA has been scarcely studied with different results [10,12,13]. Some studies concluded that ACS outcomes are worse in RA, while others showed decreased mortality of ACS in RA after adjusting for traditional CV risk factors like hypertension, diabetes mellitus (DM), tobacco, and family history of CV disease [14]. Though it is well established in the literature that RA increases the risk of CV events, there remains a scarcity of national population-based research on outcomes of ACS in RA patients. This study aims to add to the body of knowledge and fill the knowledge gap of the uncertainty of the impact of RA on outcomes of patients admitted for ACS.

## Materials and methods

Data source

We conducted a retrospective cross-sectional study of hospitalizations in 2016 and 2017 with a principal diagnosis of ACS with and without a secondary diagnosis of RA in acute-care hospitals across the United States. Hospitalizations were selected from the NIS database. The NIS was created and is maintained by the Agency for Healthcare Research and Quality and is the largest publicly available all-payer inpatient database in the United States. It was designed as a stratified probability sample to be representative of all nonfederal acute-care hospitals nationwide. Hospitals are stratified according to ownership, urban or rural location, geographic region, bed size, and teaching status. A 20% probability sample of all hospitals within each stratum is then collected. All discharges from these hospitals are recorded and then weighted to ensure that they are nationally representative. The 2016 and 2017 NIS sampling frame includes data from 47 statewide data organizations (46 states plus the District of Columbia) covering more than 97% of the U.S. population. 30 discharge diagnoses for each hospitalization can be recorded using the International Classification of Diseases, Tenth Revision, Clinical Modification (ICD-10) in NIS 2016, and as many as 40 discharge diagnoses in NIS 2017 database. In the NIS, diagnoses are divided into two separate categories: principal diagnosis and secondary diagnoses. A principal diagnosis was the main ICD-10 code for the hospitalization. Secondary diagnoses were any ICD-10 code other than the principal diagnosis. There is no reliable mechanism to distinguish secondary diagnoses that antedated the hospital admission from those that had onset during the index admission. Since all patient data in NIS are de-identified and publicly available, institutional review board approval was not sought.

Inclusion criteria and study variables

The study population consisted of all inpatient hospitalizations recorded in the NIS 2016 and 2017. Study variables included age, gender, race, hospital characteristics, medical comorbidities, primary and secondary outcomes (outlined below). We used the following ICD-10 codes to identify principal/secondary diagnoses: ACS all I21.0, I21.1, I21.2, I21.3, 121.4, I20.0 codes, and RA all M05 and M06 codes (see appendix). We studied baseline characteristics and outcomes for ACS hospitalization with and without RA.

Outcomes

The primary outcome was inpatient mortality. Rates of percutaneous transluminal coronary angioplasty (PTCA)/coronary atherectomy, percutaneous coronary intervention (PCI) with drug-eluting stent (PCI DES), PCI with bare-metal stent (PCI BMS), intra-aortic balloon pump (IABP), percutaneous external assist device (PEAD), intracoronary artery thrombolytic infusion, coronary artery bypass surgery (CABG), mean hospital length of stay (LOS) and mean total hospitalization charges were compared as secondary outcomes of interest.

Statistical analysis

Analyses were performed using STATA version 16 (StataCorp, Texas, USA). A univariate logistic regression analysis using all variables and comorbidities in Table 1 was used to calculate unadjusted odds ratios (ORs) for the primary outcome. All variables with P-values <0.1 were included in a multivariate logistic regression model. Univariate association of variables and comorbidities with the primary outcome, highlighting the variables included in the multivariable logistic regression model, are displayed in Table 2. P-values <0.05 were considered significant in the multivariate analysis. Confounders were selected from literature review. Charlson comorbidity index, which is recorded in the NIS database, was used to adjust for comorbidity burden. Multivariate logistic and linear regression model, with all variables and comorbidities in Table 1, were used to adjust for confounders for the secondary outcomes.

## Results

There were over 71 million discharges included in the combined 2016 and 2017 NIS database. Out of 1,319,464 hospitalizations for ACS, 22,615 (1.7%) had RA, while 1,296,849 (98.3%) did not have RA. Characteristics of ACS hospitalizations with and without co-existing RA are displayed in Table 1. RA group was older (70.4 vs 66.8 years, P<0.0001), and had more females (63.7% vs 37.7%, P<0.0001).

**Table 1:** Baseline characteristics of ACS hospitalizations with and without RA MI: myocardial infarction; PCI: percutaneous coronary intervention; CABG: coronary artery bypass graft; COPD: chronic obstructive pulmonary disease; DM: diabetes mellitus; CHF: chronic congestive heart failure; CKD: chronic kidney disease; O2: oxygen; A. Fib/flutter: Atrial fibrillation/flutter; median household income: median household income for patient’s ZIP code

	ACS (n=1,319,464)		
	Without RA (n=1,296,849)	With RA (n=22,615)	P-value
Mean Age (years)	66.8	70.4	<0.0001
Female	37.7%	63.7%	<0.0001
Race			<0.0001
White	73.7%	76.8%	Reference
Black	11.4%	10.8%	0.054
Hispanic	8.6%	7.5%	0.005
Asians	2.8%	1.9%	<0.0001
Native Americans	0.55%	0.60%	0.847
Others	3.0%	2.4%	0.017
Charlson comorbidity index			<0.0001
0	0.55%	0	
1	24.4%	0.55%	
2	24.7%	19.5%	
≥3	50.3%	80.0%	
Hospital bed size			0.6092
Small	16.6%	16.7%	
Medium	30.5%	29.8%	
Large	52.9%	53.6%	
Hospital teaching status			0.1962
Nonteaching	33.6%	34.5%	
Teaching	66.5%	65.5%	
Hospital location			<0.0001
Rural	7.8%	9.6%	
Urban	92.2%	90.4%	
Expected primary payer			<0.0001
Medicare	58.9%	74.3%	
Medicaid	9.8%	6.2%	
Private	26.6%	17.8%	
Self-pay	4.7%	1.7%	
Median household income (quartile)			0.7483
1^st ^(0-25^th^)	30.9%	30.5%	
2^nd ^(26^th^-50^th^)	27.5%	27.9%	
3^rd^ (51^st^-75^th^)	23.4%	22.9%	
4^th^ (76^th^-100^th^)	18.3%	18.7%	
Hospital region			0.0001
Northeast	17.9%	17.7%	
Midwest	22.4%	25.3%	
South	40.8%	39.1%	
West	18.9%	17.9%	
Dyslipidemia	66.2%	64.3%	0.0086
Old MI	15.3%	16.6%	0.0172
Old PCI	1.7%	1.8%	0.7893
Old CABG	10.2%	8.7%	0.0009
Old pacemaker	3.0%	3.5%	0.0632
A. Fib/flutter	19.6%	22.7%	<0.0001
COPD	17.9%	26.0%	<0.0001
Carotid artery disease	2.3%	2.5%	0.2685
Old stroke	1.1%	1.2%	0.5856
Hypertension	47.9%	48.6%	0.3366
Peripheral vessel disease	6.8%	7.5%	0.0532
Hypothyroidism	11.7%	19.4%	<0.0001
DM type 1 & 2	40.0%	35.8%	<0.0001
Obesity	18.2%	16.4%	0.0024
CHF	33.4%	36.8%	<0.0001
CKD	23.5%	24.7%	0.0454
Liver disease	3.4%	3.1%	0.2586
Electrolyte derangement	21.4%	23.9%	<0.0001
Maintenance hemodialysis	3.2%	2.2%	0.0003
O2 dependence	2.4%	4.0%	<0.0001
Smoking	27.2%	31.8%	<0.0001
Anemia	22.6%	29.9%	<0.0001

Univariate association of the baseline characteristics with the primary outcome is shown in Table 2.

**Table 2:** Univariate association of baseline variables with inpatient mortality MI: myocardial infarction; PCI: percutaneous coronary intervention; CABG: coronary artery bypass graft; COPD: chronic obstructive pulmonary disease; DM: diabetes mellitus; CHF: chronic congestive heart failure; CKD: chronic kidney disease; O2: oxygen; median household income: median household income for patient’s ZIP code * variable included in the multivariable logistic regression model

Baseline variables	Odds Ratio	P-value
Age*	1.04	<0.0001
Female gender*	1.17	<0.0001
Race*		
White	Reference	Reference
Black	0.81	<0.0001
Hispanic	0.91	0.013
Asians	1.21	<0.0001
Native Americans	0.83	0.174
Others	1.07	0.152
Charleston comorbidity index*	1.21	<0.0001
Hospital bed size*		
Small	Reference	Reference
Medium	1.09	0.002
Large	1.14	<0.0001
Hospital teaching status*	1.09	<0.0001
Hospital location	1.04	0.186
Expected primary payer*		
Medicare	Reference	Reference
Medicaid	0.49	<0.0001
Private	0.39	<0.0001
Self-pay	0.60	<0.0001
Median household income(quartile)*		
1^st ^(0-25^th^)	Reference	Reference
2^nd ^(26^th^-50^th^)	1.00	0.874
3^rd^ (51^st^-75^th^)	0.93	0.010
4^th^ (76^th^-100^th^)	1.01	0.713
Hospital region*		
Northeast	Reference	Reference
Midwest	0.93	0.010
South	0.98	0.385
West	1.02	0.542
Dyslipidemia*	0.44	<0.0001
Old MI*	0.78	<0.0001
Old PCI*	0.82	0.011
Old CABG	1.05	0.106
Old pacemaker*	1.17	0.002
A. Fib/flutter*	1.85	<0.0001
COPD*	1.26	<0.0001
Carotid artery disease	1.00	0.976
Old stroke*	1.49	<0.0001
Hypertension*	0.48	<0.0001
Peripheral vessel disease*	1.42	<0.0001
Hypothyroidism*	1.09	0.002
DM type 1 & 2	1.02	0.248
Obesity*	0.57	<0.0001
CHF*	2.36	<0.0001
CKD*	1.86	<0.0001
Liver disease*	7.07	<0.0001
Electrolyte derangement*	4.79	<0.0001
Maintenance hemodialysis*	1.83	<0.0001
O2 dependence*	1.36	<0.0001
Smoking*	0.83	<0.0001
Anemia*	1.67	<0.0001

60,750 adult ACS hospitalizations (4.6%) resulted in inpatient mortality. 1,010 (4.5%) of the deaths occurred in ACS hospitalizations with coexisting RA vs 59,740 (4.6%) in those without co-existing RA (P=0.6637). After adjusting for comorbidities (see Table 2) in the multivariate logistic regression analysis, hospitalizations for ACS (STEMI, NSTEMI, and UA) with RA had a 16% reduced risk of in-hospital mortality, OR: 0.84 95% confidence interval (CI) (0.72- 0.99), P=0.034, compared to those without RA. Outcomes of ACS hospitalizations with and without co-existing RA are displayed in Table 3.

**Table 3:** Clinical outcomes of ACS hospitalizations with and without RA PTCA: percutaneous transluminal coronary angioplasty; PCI DES: percutaneous coronary intervention with drug-eluting stent; PCI BMS: percutaneous coronary intervention with bare-metal stent; IABP: intra-aortic balloon pump; PEAD: percutaneous external assist devices; thrombolytics: intracoronary artery thrombolytic infusion; CABG: coronary artery bypass graft; OR: odds ratio; CI: confidence interval; USD: United States dollars * statistically significant

	ACS with RA (n=22,615)	ACS without RA (n=1,296,849)	Adjusted OR	p-value
	%	%	(95% CI)	
Primary outcome				
In-hospital mortality	4.5	4.6	0.84 (0.72-0.99)	0.034*
Secondary outcomes				
PTCA	4.3	5.1	0.94 (0.81-1.10)	0.451
PCI DES	35.4	40.5	1.14 (1.07-1.23)	<0.0001*
PCI BMS	7.1	7.9	1.01 (0.90-1.14)	0.884
IABP	2.8	4.0	0.78 (0.64-0.95)	0.015*
PEAD	0.88	1.0	1.07 (0.77-1.48)	0.697
Thrombolytics	0.24	0.29	1.07 (0.56-2.05)	0.836
CABG	6.9	8.1	0.95 (0.83-1.09)	0.473
			Adjusted mean difference	
LOS, mean days	4.6	4.4	-0.18 (- {0.32- 0.05}	0.009*
Total charge, mean, USD	85,273	91,889	-888 ({-3845}-2070)	0.556

Hospitalizations for ACS with RA had less odds of undergoing IABP, OR: 0.78, 95% CI (0.64- 0.95), P=0.015, and 0.18 days shorter adjusted mean LOS 95% CI (0.32-0.05), P=0.009 compared to those without RA. However, the adjusted odds of undergoing percutaneous coronary intervention with drug-eluting stent (PCI DES), OR: 1.14, 95% CI (1.07-1.23), P<0.0001 was significantly higher in ACS with RA group compared to ACS without RA.

## Discussion

The main findings of our study are: 1) patients admitted for ACS with co-existing RA had less adjusted inpatient mortality and LOS compared to those without RA. 2) ACS hospitalizations with co-existing RA had greater adjusted odds of undergoing PCI DES, but less odds of undergoing IABP placement compared to those without RA. IABP is used for refractory cardiogenic shock, which is a known complication of acute myocardial infarction [15].

In our study, the RA group had less traditional CV risk factors such as dyslipidemia, DM, obesity, and maintenance hemodialysis. However, the RA group had more smokers, CHF, CKD, and atrial fibrillation/flutter. We adjusted for all these differences in the multivariate logistic model.

A large population-based study comparing five-year mortality of incident RA cases compared to the general population showed improvement in mortality of 2001-2006 cohort compared to the 1996-2000 cohort [16]. The improved outcome of RA patients after the year 2000 in this study was attributed to improved management of RA with disease-modifying anti-rheumatic drugs (DMARDS) and resultant lower underlying inflammation [16]. This is similar to a 15-year prospective cohort Dutch study which showed increased mortality (particularly for CV diseases) in RA patients compared to the general population; however, the mortality tended to decrease over time [17]. RA patients have 1.5-2.0 times increased risk of developing coronary artery disease (CAD) compared to the general population, which is similar to DM [6,18,10]. An interplay between traditional CV risk factors, chronic inflammation, and RA treatment (such as high dose corticosteroids) has been implicated in the pathogenesis of the increased CV risk in RA [19].

Most large population-based studies on RA are about the mortality risk of RA compared to the general population, and on the increased risk of CV disorders in RA. There is a scarcity of studies comparing outcomes of ACS in RA patients to ACS patients without RA. We found three national population-based studies on outcomes of ACS in RA patients with rather contrasting results.

The first study was done in Taiwan. This study showed increased in-hospital mortality of RA patients with acute myocardial infarction (MI) [20]. This may be due to the difference in availability and utilization of DMARDS for RA in the U.S. population compared to the Taiwanese population. This is supported by studies from developing countries, which showed higher mortality rates in RA relative to developed countries like the US [21].

The second study was a nation-wide population-based Swedish cohort study, which found that seven and 30-day short-term mortality after ACS was worse in RA cohort vs general population [12]. This is an older study with cohort follow-up period between January 2007 to December 2010. The results from this Swedish population study may not be generalizable to the U.S. population due to the difference in both health care systems and the sociodemographic difference between both populations. Information on known CAD risk factors such as body mass index (BMI) and smoking was not available, therefore, were not adjusted for during the analysis of this study.

Our results are more aligned with the third study, a U.S.-based population study using NIS 2002-2016 database, which showed that RA is associated with lower inpatient mortality in acute MI [22]. It was hypothesized that the use of immunotherapy had a modulatory and possibly a protective effect in the RA cohort. This study used older NIS databases and combined ICD-9 and 10 codes, which can be problematic. In our study, we used the two most recent releases of the NIS databases from the ICD-10 era.

Our study has several strengths. First, we utilized data from the most recent hospitalization database available at the US population level (NIS 2016 and 2017). Second, we had a large sample size, which would give the study a high power. Third, we exclusively used the latest ICD-10 codes, which helped to further characterize the cohort and outline comorbidities that could have been confounders in prior studies.

There are some limitations to our study. First, NIS database analysis is subject to biases of retrospective studies. Second, there is a possibility of error associated with coding, as the NIS is an administrative database that uses ICD-10 codes to identify diseases and hospitalization events. Third, most of the ICD-10 billing codes fail to grade disease severity. Thus, we cannot determine if underlying RA disease severity/active disease status affected the outcome of ACS. Fourth, NIS reports data on hospitalizations, rather than individual patients. Therefore, patients hospitalized multiple times cannot be discerned. Fifth, data on specific DMARDs used by patients, rate of use, medication adherence, as well as laboratory, and radiologic data which could indicate inflammatory activity and disease severity are lacking in the NIS database. Lastly, differences in treatment modalities (PCI DES and IABP) may be confounded by indication, rather than the presence or absence of RA. However, we hope the large sample size and improved uniformity in diagnostic and therapeutic coding would compensate for these deficiencies. More research is needed to understand the mechanism by which co-existing RA imparts outcomes of patients admitted for ACS.

## Conclusions

Patients admitted for ACS with co-existing RA had lower adjusted inpatient mortality, less odds of undergoing IABP, shorter adjusted LOS, and greater adjusted odds of undergoing PCI DES compared to those without RA. Although RA increases the risk of developing ACS, RA does not negatively impact outcomes of ACS hospitalizations based on this large U.S. national database.

## Appendices

**Table 4:** Used ICD-10 codes

	ICD-10 codes
Diagnosis codes	
ACS	I21.0, I21.1, I21.2, I21.3, 121.4, 120.0
RA	M05, M06
Procedure codes	
PTCA	02703ZZ, 02704ZZ, 02713ZZ, 02714ZZ, 02723ZZ, 02724ZZ, 02733ZZ, 02734ZZ
PCI BMS	02703D6, 02703DZ, 02704D6, 02704DZ, 02703E6, 02703EZ, 02704E6, 02704EZ, 02703F6, 02703FZ, 02704F6, 02704FZ, 02703G6, 02703GZ, 02704G6, 02704GZ, 02713D6, 02713DZ, 02714D6, 02714DZ, 02713E6, 02713EZ, 02714E6, 02714EZ, 02713F6, 02713FZ, 02714F6, 02714FZ, 02713G6, 02713GZ, 02714G6, 02714GZ, 02723D6, 02723DZ, 02724D6, 02724DZ, 02723E6, 02723EZ, 02724E6, 02724E6, 02724EZ, 02723F6, 02723FZ, 02724F6, 02724FZ, 02723G6, 02723GZ, 02724G6, 02724GZ, 02733D6, 02733DZ, 02734D6, 02734DZ, 02733E6, 02733EZ, 02734E6, 02734EZ, 02733F6, 02733FZ, 02733FZ, 02734F6, 02733G6, 02733GZ, 02734G6, 02734GZ
PCI DES	0270346, 027034Z, 0270446, 027044Z, 0270356, 027035Z, 0270456, 027045Z, 0270366, 027036Z, 0270466, 027046Z, 0270376, 027037Z, 0270476, 027047Z, 0271346, 027134Z, 0271446, 027144Z, 0271356, 027135Z, 0271456, 027145Z, 0271366, 027136Z, 0270376, 0271466, 027146Z, 0271376, 027137Z, 0271476, 027147Z, 0272346, 027234Z, 0272446, 027244Z, 0272356, 027235Z, 0272456, 027245Z, 0272366, 027236Z, 027246Z, 0272376, 027237Z, 0272476, 027035Z, 027247Z, 0273346, 027334Z, 0273446, 027344Z, 0273356, 027335Z, 0273456, 027345Z, 0273366, 027336Z, 0273466, 027346Z, 0273376, 027337Z, 027045Z, 0273476, 027347Z
IABP	5A02210, 5A02110
PEAD	02HA0RJ, 02HA3RJ, 02HA4RJ, 5A02116, 5A0211D, 5A02216, 5A0221D, 02HA3RZ, 5A02216
Intracoronary artery thrombolytic infusion	3E07017, 3E07317
CABG	0210093, 0210098, 0210099, 021009C, 021009F, 021009W, 02100A3, 02100A8, 02100A9, 02100AC, 02100AF, 02100AW, 0211093, 0211098, 0211099, 021109C, 021109F, 021109W, 02110A3, 02110A8, 02110A9, 02110AC, 02110AF, 02110A, 0212093, 0212098, 0212099, 021209C, 021209F, 021209W, 02120A3, 02120A8, 02120A9, 02120AC, 02120AF, 02120AW, 0213093, 0213098, 0213099, 021309C, 021309F, 021309W, 02130A3, 02130A8, 02130A9, 02130AC, 02130AF, 02130AW
Comorbidities	
Dyslipidemia	E78
Old MI	I252
Old PCI	Z9861
Old CABG	Z951
Old pacemaker	Z950
Atrial fibrillation/flutter	I48
Chronic obstructive pulmonary disease	J41, J42, J43, J44
Carotid artery disease	I652
Old stroke	I63
Hypertension	I10
Peripheral vascular disease	I739
Hypothyroidism	E03
Diabetes mellitus type 1 & 2	E10, E11
Obesity	E660, E6601, E6609, E661, E662, E668, E669
Congestive heart failure	I50
Chronic kidney disease	N18
Liver disease	K70, K71, K72, K73, K74, K75, K76, K77
Electrolyte derangement	E870, E871, E872, E873, E874, E875, E876
Maintenance dialysis	Z992
Oxygen dependence	Z9981
Smoking	Z87891, F17200
Anemia	D50, D51, D52, D53, D55, D56, D57, D58, D59, D60, D61, D62, D63, D64
